# Age-dependent partnering and the HIV transmission chain: a microsimulation analysis

**DOI:** 10.1098/rsif.2013.0613

**Published:** 2013-11-06

**Authors:** Anna Bershteyn, Daniel J. Klein, Philip A. Eckhoff

**Affiliations:** Institute for Disease Modeling, 1555 132nd Avenue NE, Bellevue, WA 98005, USA

**Keywords:** microsimulation, HIV, transmission, analysis, age structure, modelling

## Abstract

Efficient planning and evaluation of human immunodeficiency virus (HIV) prevention programmes requires an understanding of what sustains the epidemic, including the mechanism by which HIV transmission keeps pace with the ageing of the infected population. Recently, more detailed population models have been developed which represent the epidemic with sufficient detail to characterize the dynamics of ongoing transmission. Here, we describe the structure and parameters of such a model, called EMOD-HIV v. 0.7. We analyse the chains of transmission that allow the HIV epidemic to propagate across age groups in this model. In order to prevent the epidemic from dying out, the virus must find younger victims faster than its extant victims age and die. The individuals who enable such transmission events in EMOD-HIV v. 0.7 are higher concurrency, co-infected males aged 26–29 and females aged 23–24. Prevention programmes that target these populations could efficiently interrupt the mechanisms that allow HIV to transmit at a pace that is faster than the progress of time.

## 1. Introduction

Pharmacological prophylaxis [1,2] and expanded treatment [3] were recently added to the world's human immunodeficiency virus (HIV) prevention toolbox. Despite the optimism ignited by these findings, the immediate reach of these tools in the generalized epidemic setting of sub-Saharan Africa is limited by funding constraints and scale-up time from current programme capacity [4]. An affordable and efficient strategy for interrupting transmission has yet to be established.

The difficulty of choosing a strategy for allocating transmission-blocking interventions stems from an incomplete picture of the epidemiological drivers of generalized HIV epidemics. Several detailed agent-based models have been created to simulate generalized epidemics with heterogeneous risk [5–9]. However, no model or trial has determined why the HIV epidemic has become widespread in the general population in a subset of African countries: whether the drivers are biological or behavioural, historical or still present, immutable or open to intervention. This makes it difficult to predict which strategies will succeed at interrupting transmission.

In the light of this urgent need for characterizing epidemic drivers, more detailed models are being developed to explore the behavioural properties of the HIV contact network and the biological properties of transmission. One such model, documented here for the first time in peer-reviewed literature, is called EMOD-HIV after the family of similarly structured models developed by the Institute for Disease Modeling, Bellevue, WA.

The goal of the EMOD-HIV model is to compare a broad set of structural and parametric modelling assumptions in order to identify a set of possible explanations for the epidemic—whether they centre on behavioural drivers, biological drivers or synergies between the two. Mapping this range of possible epidemic drivers will help us identify the data required to narrow down the possibilities. It will also allow for a range of predictions about the potential impact of interventions given present-day knowledge, thus helping to quantify the structural uncertainty in model predictions.

The HIV Modelling Consortium has brought together authors of a dozen different epidemiological models, in order to perform joint modelling efforts in policy-relevant areas and to investigate the sensitivity of the outcomes to model assumptions. The first of these formal model comparisons examined a dozen models’ estimates of the potential impact of treatment as prevention [10], including results from EMOD and other individual-based microsimulations [5–7].

Unlike other models, EMOD-HIV predicted both a high cost and a greater potential impact of very early treatment as a way to avert future HIV infections. The version of EMOD-HIV used to produce these results (termed v. 0.7) made similar assumptions to other models about HIV progression rates, transmission rates and the effects of antiretroviral therapy (ART). It differed primarily in the way that the transmission network was constructed and HIV risk was distributed. We hypothesize that the age-driven heterogeneities in partnership assortativity distinguished EMOD-HIV by creating a relatively young group of ‘key transmitters’. Early ART initiation allowed for suppression of infectivity in these early years of infection, but also led to higher programme costs because of the many life-years of ART consumed by those who initiate at young ages.

This difference in model outcomes is a salient example of the importance of underlying epidemic drivers in population models of HIV. Although all the models were able to reproduce similar baseline trends such as prevalence and incidence, they responded differently to an intervention because different mechanisms were driving the simulated epidemics.

To convey these drivers in EMOD-HIV v. 0.7, we first describe the model in detail, with a complete parameter table and descriptions of the model structure. We then explore how these assumptions distribute transmission potential among the simulated agents. After an in-depth look at the transmission pathways of HIV, we identify the key transmission events through which the epidemic is able to sustain itself in younger age groups, and thus propagate more efficiently through transitory and informal partnerships. We then determine the most efficient subpopulations to target with transmission-blocking interventions, in order to break transmission chains that maintain infections in these age groups. We conclude with a hypothesis about ageing of the HIV epidemic in the face of age-based assortativity, and discuss the possible sensitivities of this outcome to model structure.

## 2. Methods

All simulations were performed using EMOD-HIV v. 0.7: a detailed agent-based model of partnership formation, HIV transmission and disease progression with and without treatment. A detailed model description is provided in the electronic supplementary material S1 in order to document the structural assumptions of this model. This supplement also shows the model's fit to national-level data disaggregated by age, sex and year. The key parametric assumptions are listed in the electronic supplementary material S2.

Briefly, the components that are described include the overall model implementation, the model's structural assumptions, the progression of a single simulation in time and our approach to model calibration. The major structural assumptions include the setting-specific demographics, individual properties, partnership formation rules and rates, partnership properties, individual-level properties, coital frequency and dilution assumptions, disease progression, transmission rates and cofactors, and mortality. A full mathematical description of the pair formation algorithm (PFA) was published recently [11], and the supplement includes a diagram and description of the algorithm.

The assumptions of EMOD-HIV v. 0.7 were not based on any preconceived notion of epidemic drivers, but instead were intended to construct a mechanistic model of HIV transmission that leverages available data. The epidemic drivers analysed in this paper were implicitly formed by the model assumptions about individual-level heterogeneities, such as age, sex, concurrency levels, circumcision status, other sexually transmitted infection (STI) status and so forth. Where possible, we used available data to parametrize individual properties, partnership formation, HIV progression and HIV transmission. When data were unavailable, conflicting or unreliable, we prioritized the corresponding parameters for calibration, choosing the parameter set that best matched population-level epidemic trends.

## 3. Results and discussion

### 3.1. Distribution and properties of high transmitters

In the electronic supplementary material, we compare outputs from EMOD-HIV with demographic and epidemiological estimates of the population and HIV prevalence in South Africa by age, sex and year. Despite agreement with other models about population and prevalence in South Africa, EMOD-HIV responds differently to interventions such as rapid scale-up of ART [10]. This is hardly surprising: though calibrated to match similar baseline trends, the different models rely on different underlying epidemic mechanisms to produce these trends. These mechanisms are embodied in assumptions about the nature of the contact network (or other form of mixing), heterogeneities among individuals (and their change over time) and transmission rates.

Because of the lack of a preconceived hypothesis about epidemic drivers, this analysis presents an opportunity to explore the drivers that are implicitly formed by a mechanistic model that implements several relevant individual-level heterogeneities. These include concurrency, STI status, male circumcision status and age to help define subpopulations that experience different levels of HIV risk.

Other than age, all of these factors were assigned independently of each other at birth. These ‘assigned’ properties impact ‘derived’ properties such as concurrency status, partner count, disease prognosis and transmission rate per contact, which, in turn, determine the transmission potential of individuals.

Age is a particularly interesting form of risk heterogeneity because it changes over time for individuals, and because its assignment of risk is indirect. Age determines the rate at which an individual is recruited into transitory, informal and marital partnerships, and therefore determines both the frequency of partnership formation and the level of assortativity with other age groups.

### 3.2. Effect of risk factors

**Figure 1.**
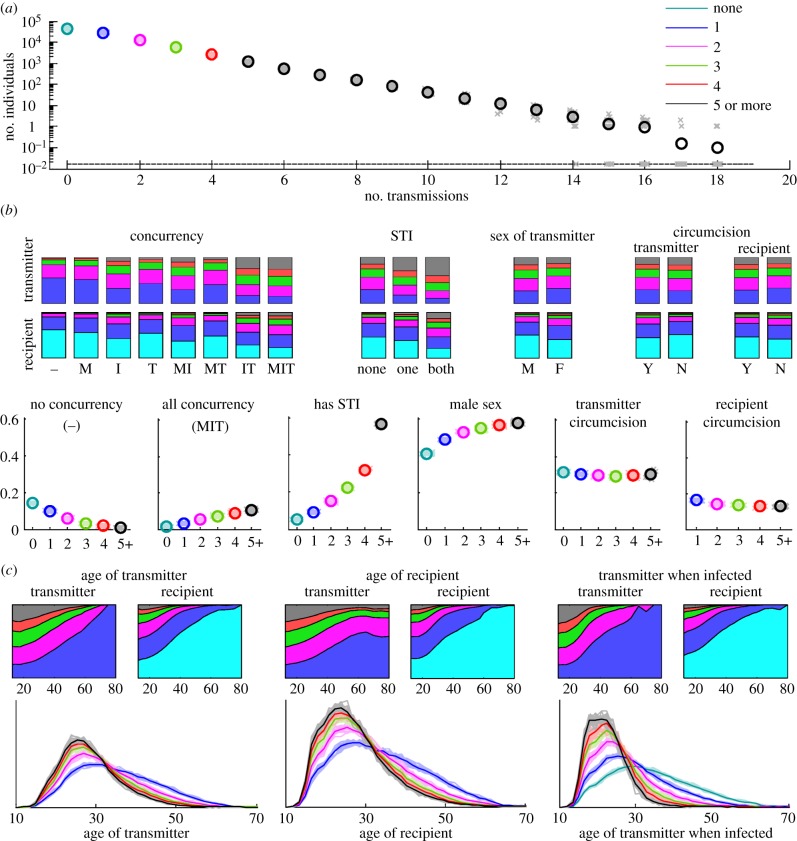
Heterogeneity in HIV transmission potential among individuals in the EMOD-HIV model. (*a*) The distribution of the number of times an infected individual transmits HIV. Faded x's show individual simulation results, whereas o's show the mean of 20 simulation results. The colours used to plot the number of non-transmitters, single-transmitters, etc. are used for the same transmitter groups in subsequent panels. Next, transmitting couples are categorized based on static properties: the transmitter's concurrency ‘flags’ (marital, informal or transitory), STI status of the transmitter or recipient, sex of the transmitter and circumcision status of a male transmitter or recipient. In (*b*), the proportions of transmitters and recipients who transmit zero, one, etc. times are shown as stacked bar charts, whereas the proportions of zero, one, etc. transmitters who have the indicated property are shown as individual simulations (x's) and means (o's). Similarly, the stacked area chart in (*c*) shows the proportion of each age group of transmitters and recipients who transmit zero, one, etc. times, whereas the curves show the age distributions for zero-, one-, etc. transmitters, with faded lines representing single simulations and dark lines showing the mean. These are shown as a function of the transmitter's age, recipient's age and transmitter's age at the time that the transmitter was originally infected.

We inspected the distribution of HIV transmission potential among agents according to their properties. This analysis is summarized in figure 1. In this analysis, individuals are stratified according to the number of HIV transmissions caused by the individual within a single simulation. This allows for an intuitive, objective measure of the ‘risk’ of transmitting HIV. The number of lifetime HIV transmissions originating from a single HIV-infected individual in EMOD-HIV ranges from zero to more than a dozen. The number of transmissions is well approximated by an exponential distribution, with roughly double the number of non-transmitters when compared with single-transmitters, double the number of single-transmitters compared with double-transmitters, and so forth (figure 1*a*).

Next, we examined the effect of concurrency ‘flags’ on transmission. Documented in the supplemental model description, these static properties allow individuals in the corresponding partnership type to acquire additional partnerships of any type, up to a maximum of six simultaneous partnerships. This ‘flag’ is common for transitory-type partnerships and rare for marital partnerships. The rate at which this potential for concurrency is realized depends on the PFA, which recruits younger individuals to transitory partnerships and older individuals to marital partnerships, with the net effect that concurrency is higher in younger individuals.

The effect of concurrency ‘flags’ on transmission is shown in figure 1*b* in two ways: the proportion of people with specific concurrency ‘flags’ who transmit once, twice, etc. times during a simulation, and the proportion of single-, double-, etc. transmitters who have specific concurrency ‘flags’. The same analysis was performed according to the concurrency ‘flags’ of the recipient of infection.

As expected, high transmitters tend to be those who were ‘assigned’ concurrency flags and transmission-enhancing STIs. For example, in the first type of analysis, individuals who became infected and had no concurrency ‘flags’ did not transmit the epidemic further in 62% of cases, and transmitted two or more times in less than 10% of cases. Meanwhile, those with all three concurrency ‘flags’ had zero forward transmissions in 23% of cases, and two or more forward transmissions in 49% of cases. In the second analysis, 14% of non-transmitters were assigned no concurrency flags, compared with 0.8% of those transmitting five or more times (figure 1*b*). Conversely, 1.6% of non-transmitters harboured all three concurrency flags, compared with 11% of those transmitting five or more times.

Similarly, transmission-enhancing STIs were over 10-fold less prevalent in non-transmitters (5.3%) than in 5+ transmitters (57%). High transmitters were more likely to be male: 41% of non-transmitters were male, compared with 58% of 5+ transmitters. Circumcision, despite protecting against 60% of transmissions, exhibited little variation among the different classes of transmitters, because the modelled effect of circumcision was on acquisition rather than on transmission, and thus had no impact on the transmission potential of already infected males.

We also examined the proportion of female transmitters who infected circumcised males, as a fraction of total female transmitters in each class. Although circumcision reduces acquisition rates, there was little difference in the proportion of circumcised partners among female single-transmitters versus 5+ transmitters. Similarly, the proportion of recipients who had STIs or particular concurrency flags varied little by the class of transmitters who infected these recipients, because these attributes were implemented as static flags without correlation between partner choice and STI or circumcision status.

The absence of variability in the recipient's risk factors as a function of transmission count can be explained by the lack of relationship assortativity by these risk factors in EMOD-HIV. Individuals with STIs, high concurrency and other risk factors had no increased probability of pairing with other individuals with similar STIs, concurrency and so forth. Owing to the paucity of data defining such assortativity properties, a range of assumptions about risk-based assortativity could be explored. It is important to note, however, that, in a network-based model such as EMOD-HIV, such assortativity does not arise naturally as a result of heterogeneous HIV risk; it must be explicitly defined as a pair formation rule in order to be observed in the resulting HIV transmission chains.

Similar risk stratifications can be explored using derived rather than assigned properties in the model. For example, the number of recent or lifetime partnerships, though not directly specified, are a direct consequence of assigned rules about concurrency and rates of recruitment into PFAs. In the electronic supplementary material, figure S6, the number of transmissions coming from individuals with one, two, three, four or five or more lifetime partners or recent partners is shown, as well as the number of transmissions coming from those infected by such individuals. Again, because there is no assortativity by partner number, transmission risk grows strongly with the transmitter's partner count, but the recipient's transmission risk does not grow with the transmitter's partner count.

### 3.3. Effect of age

Age was the only ‘assigned’ property for which pair formation obeyed assortativity rules, which were dictated by the data-driven matrices of partnership age distributions (MOPADs). As shown in figure 1*c*, high transmitters tended to be younger at the time they caused transmissions. Owing to assortativity by age, the partners they infected also tended to be younger. Predictably, high transmitters were not only younger at the time they transmitted to others, but also younger at their time of infection, relative to low and non-transmitters. The proportion of individuals of each age who transmitted a given number of times is shown in the electronic supplementary material, figure S1, and reveals a similar trend.

**Figure 2.**
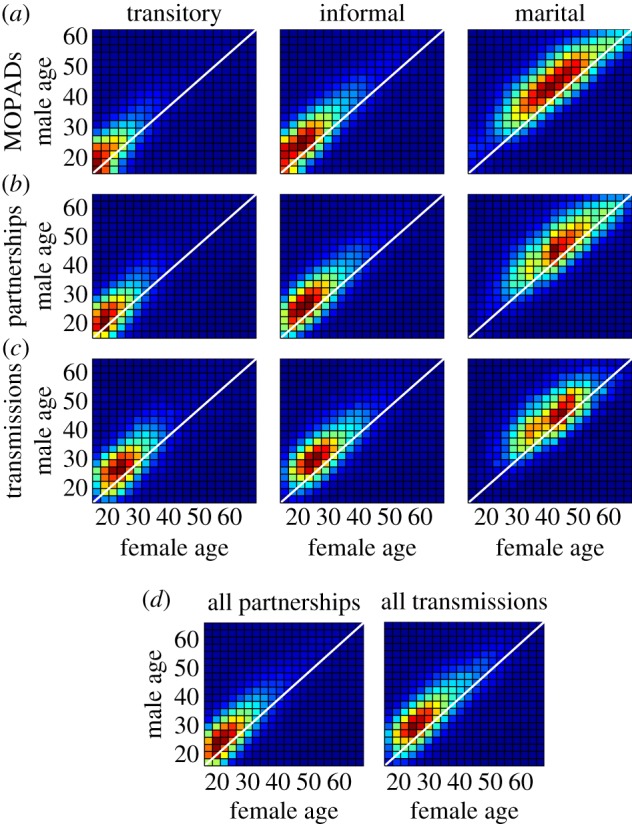
Comparison of age-mixing patterns used for model input, realized as model output, and occurring during transmission of HIV. A MOPAD (*a*) is used as input for the PFA forming each partnership type. On average, transitory relationships recruit the youngest individuals, create the smallest age gap between partners and have the shortest durations, whereas marital partnerships recruit the oldest individuals, create the largest age gap between partners (approx. 5 years) and have the longest durations. The age-mixing pattern upon formation of these partnerships (*b*) is close to the MOPADs, but with added noise reflecting the stochastic nature of the simulation. The age pattern of couples at the time of HIV transmission (*c*) is older, as HIV is transmitted after partnerships have formed. This age lag can also be seen after averaging across the three types (*d*).

The MOPADs that dictate these age-mixing patterns are shown in figure 2. The input MOPADs (figure 2*a*) are similar to the realized age pattern among relationships formed in the model (figure 2*b*), except that the model output includes some noise owing to stochastic fluctuations in the randomly formed partnerships. This verifies that the PFA is able to form partnerships according to the MOPADs. The age pattern of transmitting couples at their time of transmission (figure 2*c*) is, predictably, older than that of partnerships upon formation, because significant time often elapses in a partnership prior to transmission of HIV.

These patterns provide some initial insights into the ages at which HIV transmission occurs in the heterosexual network. To further our understanding of these patterns, we next explore HIV transmission in the context of the overall network and the age dynamics of the population.

### 3.4. Chains of transmission

The history of transmission can be seen as a tree, rooted in one of more introductions of HIV into the population, growing forward in time when an individual infects another, and branching when one transmitter infects multiple recipients. This perspective allows us to further investigate the role of age-based risk from both an individual-level perspective and a simulation-wide transmission ‘tree’ perspective.

**Figure 3.**
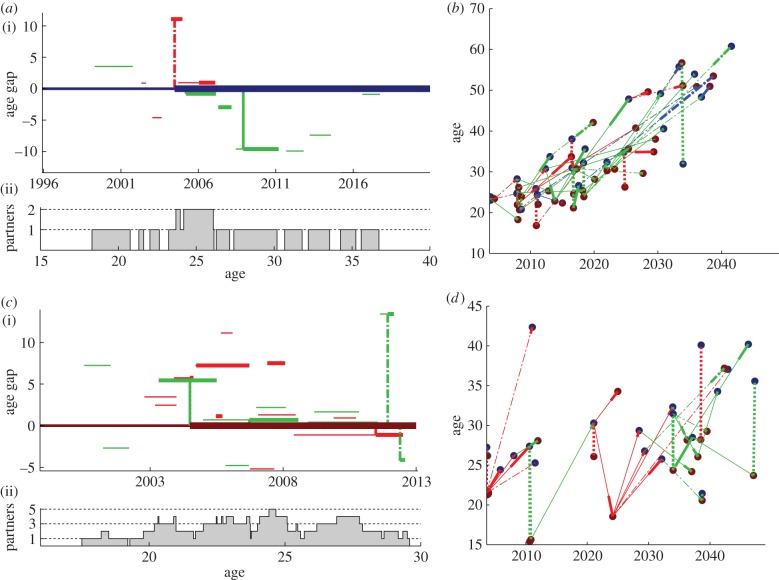
Sample lifecourses and chains of transmission. (*a,c*) A lifetime of relationships and HIV transmission from the perspective of a male and female individual, respectively. (i) The relationship gaps between the individual (darker line at zero) and their transitory (red), informal (green) and marital (blue) partners. Horizontal lines become thick when individuals become infected. Vertical lines indicate transmission events during the acute (dotted lines), latent (solid lines) or acquired immunodeficiency syndrome (AIDS) (dashed-dotted lines) stage. Note that the partners can become infected from outside the relationship shown. (ii) The number of simultaneous partnerships against the individual's age. (*b*,*d*) The tree of downstream transmissions that originate in the root individual in (*a*) and (*c*), respectively. Transmission events are plotted at the time of transmission and age of the receiving individual. Connecting lines show the type of relationship causing the upcoming transmission (red, transitory; green, informal; blue, marital) and the stage of disease during transmission (dotted, acute; solid, latent; dashed-dotted, AIDS).

An individual can be viewed as a timeline of relationship formation, infection and onward transmission, as shown in figure 3*a,c* from the perspective of a male and female individual, respectively. In figure 3*a*, the male has an extra-transitory flag (which 80% of individuals are given in the model), allowing him to acquire additional partnerships while already involved in one or more transitory partnerships. The female in figure 3*c* has both the extra-transitory and the extra-informal flag, a property shared by 24% of individuals in the model. Both individuals’ numbers of lifetime partners are relatively high, allowing us to see multiple forward transmissions of HIV.

The relatively high partner counts are in part attributable to the lack of marital partnership formation in these two individuals. As discussed earlier, extramarital flags are relatively rare and marriages are relatively long, reducing the additional partnerships taken on by individuals after marriage. (The risk of transmission in a discordant marriage, however, is relatively high owing to its long duration and lower rate of condom usage.)

Figure 3*b,d* shows the tree of transmissions that can be traced back to the ‘root’ individual in figure 3*a*,*c*, respectively. Note that eliminating infections from the root individual would not necessarily have prevented all these individuals from becoming infected, as they could have been infected later by a different individual—a superinfection event that would have become a primary infection if the transmission from the root had been blocked.

In figure 3*b*, it is striking that the ages at the time of infection increase over time. This ‘ageing’ of the infection can occur even with negative age gaps, if the time interval between transmission events is greater than the age gaps between partners. For example, if an individual is infected at age 20 and transmits the infection 5 years later, then the partner must be more than 5 years younger in order to receive a ‘non-ageing’ infection. Examples of such ‘non-ageing’ transmissions can be seen in figure 3*d*.

### 3.5. Non-ageing transmissions

From examples of transmission trees, we have shown how non-ageing transmissions (in which the transmitter was older than the recipient at their respective infection times) maintain the epidemic in young age groups. What follows is a quantitative characterization of these transmission events.

**Figure 4.**
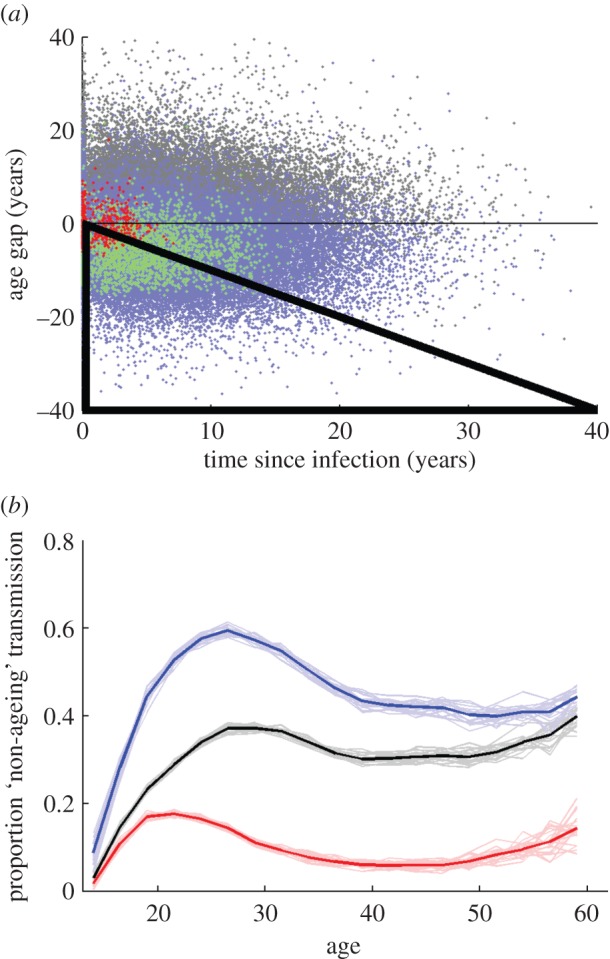
Proportion of transmissions that are non-ageing. (*a*) Scatter plot of all transmission events in a simulation, showing the years since the transmitting individual was infected (horizontal axis) and age gap between the partners (vertical axis). Transmissions that fall within the black triangle are non-ageing, i.e. when the recipient is younger than the transmitter at their respective infection times. Overlaid blue points are male-to-female transmissions only; green points are transmissions from 30-year-old males; red points are transmissions from 20-year-old males. (*b*) Proportion of male-to-female (blue), female-to-male (red) and all (black) transmissions that are non-ageing, by age of the transmitter. Faded lines show individual simulations. Dark lines show the mean of 20 simulations. Dashed lines show 1 s.d. from the mean, which diverges from the mean as the number of transmissions increases to numbers that are more rare.

We begin by analysing the distribution of partnership age gaps relative to the time intervals between infection and onward transmission. Non-ageing transmission events require a negative age gap (transmitter older than recipient) that is greater than the time since infection of the transmitter. In a scatter plot of time since infection versus age gap in figure 4*a*, non-ageing transmissions lie below the diagonal line that forms the black triangle. Overall, one-third of transmissions in a simulation are ‘non-ageing’, falling inside the triangle.

Although the cloud of all transmissions is symmetric about the horizontal axis, the subset of male-to-female transmissions (blue dots) is more likely to have a negative age gap: 5% of male-to-female transmissions are non-ageing, compared with 15% of female-to-male transmissions.

Age influences both the average partnership age gap and the average time between transmissions. For example, the red dots in figure 4*a* show transmissions from 20-year-old males, and the green dots show transmissions from 30-year-old males. Transmissions from the 30-year-old males land further out along the horizontal axis, causing some of them to fall outside the non-ageing triangle, but they are also more dispersed along the vertical axis, with a pronounced and sex-biased age gap, enabling male-to-female transmissions to fall within the non-ageing triangle.

### 3.6. The ‘tug-of-war’ of an ageing epidemic

As we have already discussed, younger individuals are recruited more frequently by the transitory and informal PFAs, leading to higher concurrency and higher rates of partner turnover, and consequently shorter gaps of time between transmissions. These MOPADs, however, tend to pair individuals of similar age, whereas the marital MOPAD, recruiting older individuals, creates the largest age gaps. This creates a ‘tug-of-war’ between two opposing effects: older individuals form larger age gaps in partnerships, but also tend to exhibit longer times between transmission events.

The outcome of the ‘tug-of-war’ is shown in figure 4*b*: the proportion of forward transmissions that are non-ageing, as a function of the age of the transmitter. This proportion is highest in 26-year-old males (blue curves) and in 21-year-old females (red curves).

Non-ageing transmissions are overall more likely to come from males than from females owing to the MOPAD-driven sex bias in partner age gaps. Thus, HIV may infect a younger individual when transmitting from a male to a female, but the next female-to-male transmission event could negate this non-ageing by infecting a new male who is older than the original male in the chain. The long-term effect would be a transmission chain that traces a zigzag, increasing in age. To study the overall ageing of the epidemic in a transmission chain, independent of sex biases in partner age, we will now focus our discussion on non-ageing round trips (NARTs).

### 3.7. Non-ageing round-trip transmissions

**Figure 5.**
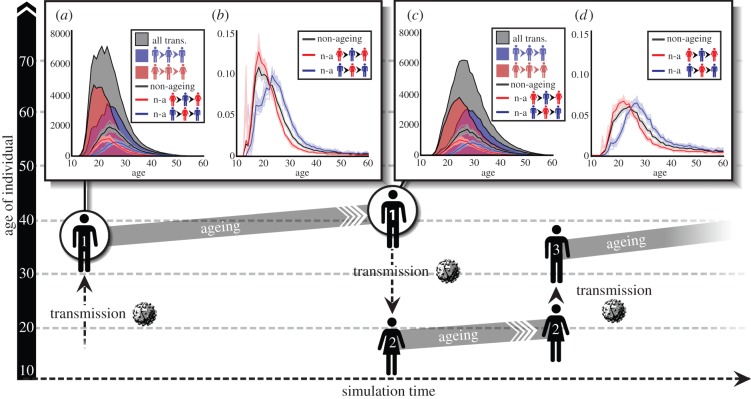
Number of round trips, NARTs and NARTs per infectious person-year, as a function of age. The diagram illustrates a possible non-ageing male–female–male round trip, with time represented on the horizontal axis and age on the vertical axis. (*a*) The total number of round-trip transmissions in a simulation, as a function of the age of the first individual in the round-trip chain at his/her time of infection (grey-shaded area). These are disaggregated into male–female–male round trips (blue area) and female–male–female round trips (red area). Below the shading, lines show the subset of these round trips that are non-ageing: male-to-female (blue), female-to-male (red) and all (black). (*b*) Number of NARTs (of the specified age and sex, as in (*a*)) per infected sexually active person-year at the sex and age of the first individual's time of infection. (*c*) Number of total round trips and NARTs, as in (*a*), as a function first individual's age at the time of *transmission*. (*d*) NARTs per infectious person-year, as in (*c*), as a function of the first individual's age at the time of transmission. Person-years are also calculated for the age at transmission. In (*a*–*d*), faded lines show individual simulations and dark lines show the mean of 20 simulations.

Round trips are defined as transmission chains from a male to a female and back to another male, or from female to male to another female. Note that the term ‘round trips’ refers to infection returning to the sex of origin, not the individual of origin. Round trips are considered non-ageing if the first individual's age at infection is older than the third individual's age at infection. The diagram in figure 5 illustrates a possible male–female–male NART.

In this analysis, we exclude round trips in which the first individual was seeded with HIV as part of the initialization of the simulated epidemic, in order not to bias the analysis by the chosen ages of the seed individuals. This excluded 1.5% of round-trip transmission chains from analysis, all of which originated during the early epidemic.

We independently counted every round trip leading to a unique third individual, even if the first one or two individuals are the same. For example, if the second individual in the round trip infects two people during the simulation, the analysis would count two round trips beginning with the same first and second individual, but ending in different third individuals.

Figure 5*a* shows the total number of round trips in a simulation as a function of the age of the first individual when initially infected. Below these shaded areas, it shows the subset of these that are non-ageing. Figure 5*c* shows the same round trips and NARTs, but as a function of the first individual's age when transmitting to the second individual.

Intuitively, one may think that half of transmission events must infect younger individuals in order to sustain an epidemic. However, this is not the case for the simulated HIV epidemic in EMOD-HIV. Overall, non-ageing transmissions constitute fewer than 40% of one-way transmissions (figure 4*b*) and fewer than 30% of round trips beginning and ending in the same sex (figure 5*a,c*).

The mechanism by which the epidemic is sustained under these circumstances is illustrated in figure 3*d*. In this tree of transmissions, a small number of non-ageing transmissions give rise to a cascade of ageing transmissions. Each chain of ageing transmissions has the potential to eventually age out of the population, as in figure 3*b*, but is ‘rescued’ by another non-ageing transmission, allowing the process to continue.

Because round trips start and end in individuals of the same sex, the number of non-ageing male–female–male and female–male–female round trips is approximately equal: hence the equal areas under the blue and red curves in figure 5*a* and, similarly, in figure 5*c*.

Round trips that begin in males are more likely to begin in an older individual, pass through a younger individual, and then return to an older individual, as in the diagram in figure 5. This explains why female–male–female round trips begin in individuals who are younger when first infected (figure 5*a*) and when transmitting (figure 5*c*) when compared with male–female–male round trips.

For example, the greatest number of round trips begin with females initially infected between ages 18 and 21 (figure 5*a*, red-shaded area), and transmitting at age 24 (figure 5*c*, red-shaded area). By contrast, the ages for which the greatest number of male-initiated round trips begin are 23–25 years at initial infection (figure 5*a*, blue-shaded area) and 27–29 years at transmission (figure 5*c*, blue-shaded area). These age ranges, for their respective sexes, represent the optimal window of time to intervene in order to prevent the initiation of a round-trip transmission.

The optimal age to prevent an NART is slightly older than the age for preventing round-trip transmission chains overall, because the youngest individuals are unlikely to find even younger individuals with whom to partner. The greatest number of overall round trips begin in females initially infected at age 23–24 (figure 5*a*, red lines), and transmitting at age 24 (figure 5*c*, red lines). For males, this age interval for interrupting the greatest number of round trips (ageing or not) is age 26 at infection (figure 5*a*, blue lines) and age 29 at transmission (figure 5*c*, blue lines).

### 3.8. Using antiretroviral therapy to reduce non-ageing round trips

From the standpoint of intervention cost-effectiveness, it may be most helpful to know the number of NARTs initiated in a given age group, per infectious person-year that fall within that age group. This accounts for the population of a given age group in the denominator of the calculation, in order to measure the efficiency of providing a transmission-blocking intervention for which cost scales with duration, such as suppressive ART.

The number of initiators of NARTs of a given sex and age at infection, per infectious (i.e. sexually active and HIV-infected) person-year of that age and sex in a simulation is shown in figure 5*b*. Similarly, the number of NARTs for which the first transmission occurs at a given age and sex, per infectious person-year at this age and sex, are shown in figure 5*d*. Based on this analysis, the most efficient age range for preventing NARTs in females is approximately age 18–22, and in males is age 23–28.

The spike in NARTs per person-year in figure 5*b*, which occurs only in a subset of the 20 simulation results shown, is due to small numbers in the denominator of infected person-years of a given age and sex. In EMOD-HIV v. 0.7, it is rare for sexual debut, let alone infection, to occur as early as age 14. When the number of person-years in the denominator fluctuates near zero in the subset of stochastic simulations, the resulting ratio fluctuates widely, although the mean is small.

### 3.9. The evolving epidemic

**Figure 6.**
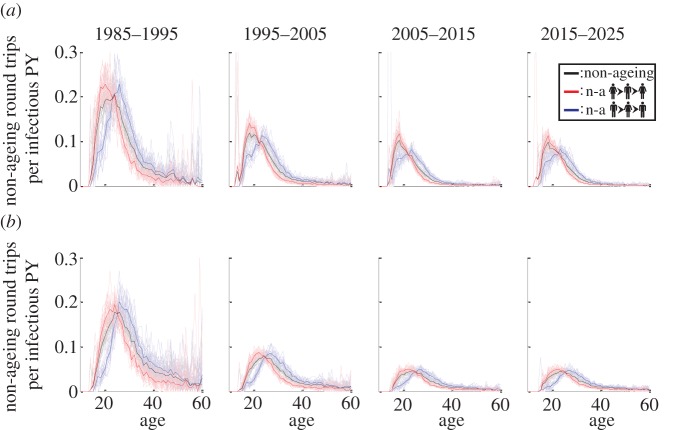
NARTs by decade. The number of male-to-female (blue), female-to-male (red) and all (black) NARTs per infectious person-year (PY) (of the corresponding age and sex) are plotted as a function of the first individual's age. In (*a*), age (horizontal axes) and person-years (denominator for vertical axes) are calculated at the age of the first individual during his/her initial infection. In (*b*), these are calculated at the age of the first individual during transmission. The non-ageing transmission chains are subdivided by decade according to the date of the first transmission. Faded lines show individual simulations and dark lines show the mean of 20 simulations. Although more transmissions per person-year occur in the earlier epidemic, the age pattern remains constant over time.

The analysis in figure 5 combines all transmission events over the course of an epidemic. However, the elements of this calculation, including age structure of the infected population and incidence rates across age groups, can vary over time. We therefore performed this analysis for different time periods of the simulation, shown in figure 6. As in figure 5*b*, spikes in the number of NARTs per person-year are seen at early ages owing to small numbers in the denominator of infected person-years of a given age and sex.

In 1985–1995, the epidemic grows rapidly, spreading from the initially infected subpopulation to a broader population. Because incidence is overall higher, the incidence of non-ageing transmission chains is also higher than in later years. In 1995–2005, condom usage increases, especially in transitory and informal partnerships, and the incidence rate declines. The 2005–2015 interval is characterized by a generalized, mature epidemic, with prevalence reaching its peak and incidence declining.

Despite these changes, the age range that produces the greatest number of NARTs remains relatively constant throughout the epidemic: ages 18–22 in women and 23–28 in men. If no major preventative programmes are rolled out, the model predicts no change in this age range in the next decade.

## 4. Discussion

We have documented the structure and parameters of a mechanistic individual-based model of heterosexual HIV transmission, and analysed the heterogeneities in transmission that result from our model assumptions. Characteristics such as circumcision, STIs and concurrency capabilities influenced transmission potential in intuitive ways.

Age, however, was a particularly interesting dimension for heterogeneity because of its change over time, its link to demographics and disease progression rates, and the complex assortativity dictated by data about partnership formation. Therefore, we chose an age-based analysis to best expose the dynamics of HIV transmission that result from our model assumptions.

Our results indicate the existence of implicit heterogeneities produced by age structuring. When compared with models that were calibrated to similar epidemic data but lack age structure (and may or may not stratify the population into risk groups), our age-structured model responded differently to the hypothetical intervention of universal ART. EMOD-HIV predicted a higher impact of very early treatment (within 1 year of infection), but also a higher cost of such a programme. The effect of treatment as prevention in EMOD-HIV was not only a decline in incidence, but also an increase in the age of infected individuals.

Based on the analysis of age-based heterogeneities presented here, we hypothesize that the early ART initiation was highly effective because it permitted treatment initiation at young enough ages to reach the key transmitters in our model. Ongoing model comparison work through the HIV Modelling Consortium will explore this hypothesis by investigating the points of divergence between EMOD and other models as a result of its assumptions.

We have provided an important starting point for this analysis by documenting the assumptions of EMOD-HIV and identifying the mechanism of epidemic propagation that results from these assumptions. Having understood the epidemic drivers that are created implicitly by the structure of EMOD-HIV, we can now compare these with structurally different models, to understand why models produce disparate predictions about the HIV epidemic.

This analysis has focused on a baseline scenario with no interventions such as ART, and the future projections of this analysis are under the assumption of no large-scale rollout of preventative interventions over the coming decade. While it is likely that future programmatic change and expansion will influence the dynamics of the HIV epidemic, there is considerable uncertainty as to the properties, scales and time courses of these programmes. Exploration of the range of these possible programmes and their impacts has been left for a future study, because of the considerable scope of this analysis. This limitation of the current analysis is important to consider, however, when placing the current analysis in the context of an expanded programme.

The analysis presented here is also limited to the stated assumptions, such as the models of within-host progression and infectivity, PFAs and concurrency, and demographics. These were the assumptions used to produce results for the model comparison published in 2012. However, these assumptions are not the only ones that are consistent with the known mechanisms and measurements of the epidemic, nor are they the only modules available for EMOD-HIV. Future work will explore the impact of changing PFAs, within-host disease staging and transmission mechanisms.

For example, these is strong evidence that high viral load is associated with both shorter survival time [12–14] and increased transmission rate [15–17]. Like the majority of models compared in the 2012 analysis, EMOD-HIV determines per-contact transmission rate as a function of disease stage. Although the duration of the latent stage is age-dependent, there is no correlation between survival time and per-contact transmission rate in the model. We hypothesize that adding this correlation would reduce the epidemiological importance of younger transmitters, because their long survival time would be offset by lower per-contact transmission rates.

The long-term goal of EMOD is to map the range of possible epidemic drivers that are consistent with measurable properties of the epidemic, but use different structural and parametric model assumptions. This analysis is necessary to identify strategies for targeting interventions, quantify the uncertainty in programme impact, and plan future studies that would best reduce these uncertainties.

## 5. Conclusion

Interrupting HIV transmission in the generalized epidemic setting of sub-Saharan Africa will probably require scale-up of HIV prevention/treatment programmes beyond their current capacity.

In a systematic comparison with other models, EMOD-HIV v. 0.7 predicted higher cost and higher impact of early ART for preventing HIV transmission. We hypothesize that this was driven by its structural assumptions, particularly about age-based relationship formation rates and assortativity.

In EMOD-HIV, high transmitters are more likely to be male, co-infected with an STI and able to form concurrent partnerships. Through examples of simulated transmission trees over time, we observed the importance of traversing age gaps, either by rapid transmission over small age gaps or by slower transmission over larger gaps. We analysed the rates of non-ageing transmissions, observing a ‘tug-of-war’ between younger individuals, who are more likely to transmit rapidly after entering high-concurrency relationships, and older individuals, who are more likely to form partnerships with larger age gaps. In this ‘tug-of-war’, the intermediate ages of 26 (for males) and 21 (for females) produced the highest proportion of non-ageing transmissions. However, the sex asymmetry in partner age meant that the epidemic could still ‘zigzag’ into older age groups by entering a younger individual when transmitting from a male to a female, but negating this age jump when entering a still older male in the next female-to-male transmission.

To circumvent the sex asymmetry in which male-to-female transmissions are more likely to be non-ageing, we analysed round-trip transmission chains, i.e. transmission from a male to a female and back to another male, or from a female to a male and back to another female. Again, the ‘tug-of-war’ created an intermediate age in which the highest number of non-ageing transmission chains were observed. We divided this count by the number of infectious person-years in the same age group to calculate the rate that infected individuals of a given age initiate NART transmissions.

We found that the maximum number of NARTs per infected person-year were initiated by males aged 26–29 and females aged 23–24. The age range of these windows of opportunity remained constant over the course of the simulated epidemic. We hypothesize that these would be the most efficient groups in which to initially provide a transmission-blocking intervention, in order to minimize NARTs.

This is, however, only one possible hypothesis about drivers of the HIV epidemic, constructed through the model assumptions that we have described. Ultimately, a range of plausible assumptions and their underlying epidemic mechanisms must be explored in order to quantify model uncertainties, inspire research and ultimately plan more efficient intervention strategies.
